# Enzymatically Polymerized Organic Conductors on Native Lipid Membranes

**DOI:** 10.1021/acs.langmuir.4c03373

**Published:** 2024-12-17

**Authors:** Diana Priyadarshini, Tobias Abrahamsson, Hanne Biesmans, Xenofon Strakosas, Jennifer Y. Gerasimov, Magnus Berggren, Daniel T. Simon, Chiara Musumeci

**Affiliations:** † Laboratory of Organic Electronics, Department of Science and Technology, Linköping University, 60174 Norrköping, Sweden

## Abstract

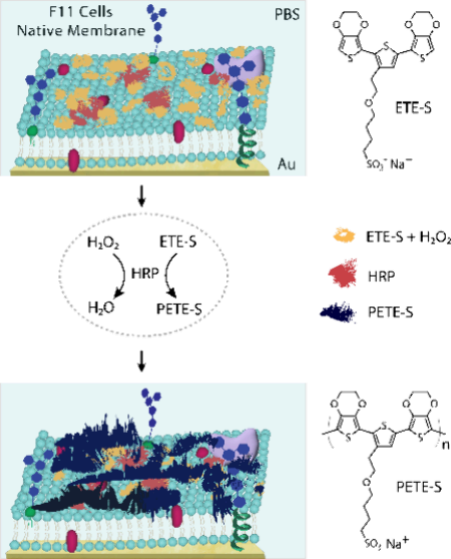

The dual capability of conductive polymers to conduct ions and electrons, in combination with their flexible mechanical properties, makes them ideal for bioelectronic applications. This study explores the *in situ* enzymatic polymerization of water-soluble π-conjugated monomers on native lipid bilayers derived from the F11 cell line, mimicking mammalian neural membranes. Enzymatic polymerization was catalyzed using horseradish peroxidase (HRP) in the presence of oxidant hydrogen peroxide (H_2_O_2_) and monitored via electrochemical quartz crystal microbalance with dissipation (EQCM-D) and electrochemical impedance spectroscopy (EIS). Results showed polymerization with HRP. The structural properties of the formed polymer films were characterized *ex situ* using atomic force microscopy (AFM), while the quality of the F11 native lipid vesicles and bilayer was respectively assessed through dynamic light scattering (DLS) and fluorescence recovery after photobleaching (FRAP) techniques. This work demonstrates, for the first time, the feasibility of the *in situ* formation of conductive polymers on native lipid membranes, offering a promising approach for the development of minimally invasive neural electrodes to diagnose and treat neurological disorders.

## Introduction

**Figure 1:**
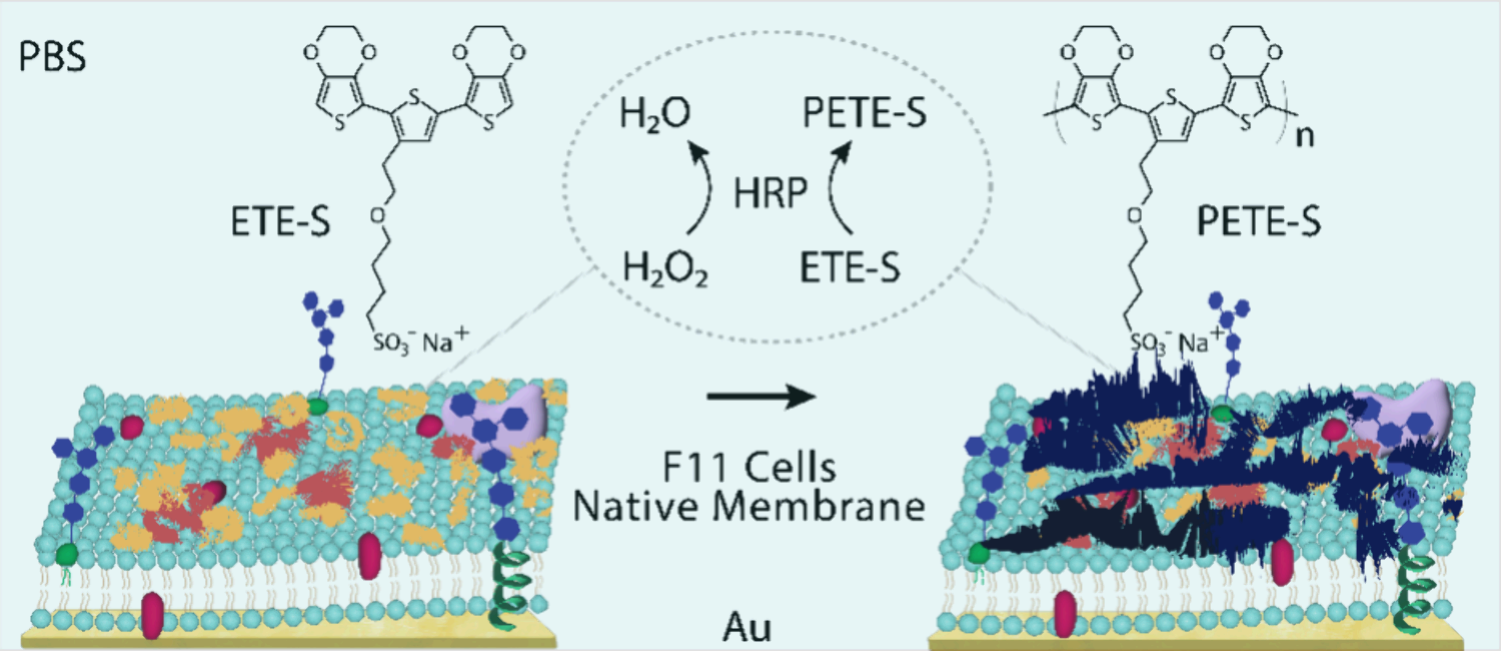
Schematic of the fluid-mosaic lipid membrane model made up of F11 cell membrane patches supported on a Au substrate, showing the ETE-S monomer (yellow) polymerized to form PETE-S (black) catalyzed by the enzyme HRP (orange) in the presence of H_2_O_2_ as oxidant, all in a phosphate buffered saline (PBS) medium; the chemical structures of the monomer and polymer are also shown.

The dual property of conductive polymers to conduct both ions and electrons, along with their lower elastic modulus compared with metallic and semiconducting materials, makes them well-suited for *in situ* as well as *in vivo* bioelectronic applications. Published works show that electrodes can be synthesized in and around living neural cells using electropolymerization or enzymatic polymerization of precursors of widely researched electroactive polymers like polyethylene dioxythiophene (PEDOT) and polyaniline (PANI).^1−3^ In particular, the water-soluble organic conducting monomer with a 2,5-bis(2,3-dihydrothieno[3,4-*b*][1,4]dioxin-5-yl)thiophene (ETE, i.e., ethylenedioxythiophene-thiophene-ethylenedioxythiophene) backbone, functionalized with a sodium 4-ethoxy-1-butanesulfonic acid salt side-chain on the central thiophene molecule (ETE-S, Figure 1), was enzymatically polymerized using oxidases and peroxidases to fabricate bioelectrodes in organisms like hydra, zebrafish, and medicinal leeches.^4−6^ The aim of these efforts is to develop novel diagnostic and therapeutic approaches for neurological disorders based on the injection of small and biocompatible (thus the advantages of water-solubility) molecule precursors which could be capable of crossing the blood–brain barrier (BBB) and forming conductive pathways through *in vivo* polymerization, thereby potentially preventing the need for invasive surgical implants altogether.^7^ Therefore, understanding the interactions of these monomer precursors with lipid bilayers, which make up basic cell membrane structures, is essential for the development of next-generation neural electrodes.

In a previous study, the effect of enzymatic polymerization of ETE-S on synthetic lipid bilayers made up of 1,2-dioleoyl-3-trimethylammonium-propane (DOTAP) was proven using the enzyme horseradish peroxidase (HRP) as the catalyst in the presence of oxidant hydrogen peroxide (H_2_O_2_).^8^ Although supported lipid bilayers (SLBs) formed using synthetic lipid vesicles provide a simple, standardized platform that is compatible with various surface characterization techniques, they pose significant challenges related to the integration of membrane proteins and are thus not sufficient for translating methods and technologies from *in vitro* to *in vivo* and ultimately to healthcare applications. Instead, SLBs formed using native lipid vesicles preserve the mobility and orientation of membrane proteins found in the native lipid environment and can therefore act as a much higher fidelity mimic of the *in vivo* cell membrane from which they are derived.^9^ As such, enzymatic polymerization of ETE-S is demonstrated in this work using native lipid membranes derived from the F11 cell line, which is a hybrid obtained by fusing N18TG2 cells from mouse neuroblastoma with dorsal root ganglion (DRG) neurons from rat embryos, and commonly employed to simulate post-differentiated sensory neurons.^10−12^

The chemically induced plasma membrane vesiculation or “blebbing” procedure is known to produce “blebs” or giant plasma membrane vesicles (GPMVs) which contain native plasma membrane proteins and physiological lipid complexity, and represent the cell membrane in a condition of thermodynamic equilibrium.^13,14^ Adsorption of such blebs derived from F11 cells on the surface of a Au/Ti coated quartz sensor and subsequent formation of a SLB, followed by the adsorption and enzymatic polymerization of ETE-S catalyzed by HRP in the presence of H_2_O_2_, were monitored *in situ* using simultaneous electrochemical quartz crystal microbalance with dissipation (EQCM-D) and electrochemical impedance spectroscopy (EIS) (Figure 1). This combination of powerful surface characterization techniques provided useful information about the structural and electrical film properties of the resulting poly(ETE-S) (PETE-S) polymer, as well as the interactions at the polymer–membrane interface. The quality of the blebs and the bleb bilayer were assessed *ex situ* through dynamic light scattering (DLS) and fluorescence recovery after photobleaching (FRAP) using confocal microscopy. The surface of the aqueous polymer–membrane system was also characterized *ex situ* using atomic force microscopy (AFM) to obtain surface morphology and topography information. Additionally, the temperature control system of the QCM-D instrument allowed the monitoring of these enzymatic polymerization reactions at both room and body temperatures. This represents the first example to date of *in situ* formation of a conducting polymer on a native lipid membrane representing a substitute model for mammalian cell membranes.

## Materials and Methods

### Chemicals and Reagents

Synthesis of ETE-S was performed as per published protocol.^15^ Horseradish peroxidase (HRP, Type I) and phosphate buffered saline (PBS) tablets were purchased from Sigma-Aldrich. Enhanced K-Blue solution containing 3,3′,5,5′-tetramethylbenzidine (TMB) and H_2_O_2_, along with a 1 N (0.5 M) H_2_SO_4_ stop solution, were purchased from NEOGEN. DOTAP lipids (10 mg/mL in CHCl_3_) were purchased from Avanti Polar Lipids. Ammonium hydroxide (NH_4_OH, 25% solution) was purchased from EMSURE, and a 35 wt % H_2_O_2_ solution in H_2_O was purchased from Acros Organics. Stock solutions were prepared at a concentration of 1 mg/mL (lipids, monomer, and HRP) or 1 M (H_2_O_2_) in PBS (10 mM phosphate buffer, 137 mM NaCl, and 2.7 mM KCl, pH 7.4 at 25 °C), from which the required diluted aliquots in PBS were freshly prepared prior to each measurement day. All measurements were conducted at the room temperature of 22 °C unless specified otherwise.

### Cell Culture, Seeding, and Blebbing

The F11 cell line was a generous gift from Dr. Michel Pohl (Université Pierre et Marie Curie 6, Paris, France) and Prof. Camilla Svensson (Karolinska Institute, Stockholm, Sweden). Cells were cultured in Ham’s F-12 Nutrient Mix GlutaMAX medium (Invitrogen) and supplemented with 15% heat inactivated fetal bovine serum (FBS, Invitrogen), 1× sodium hypoxanthine, aminopterin, and thymidine (HAT, Invitrogen), 1× penicillin/streptomycin (Invitrogen), and allo-4-hydroxy-l-proline 200 μg/mL (Sigma-Aldrich). The F11 cells were grown in a humidified atmosphere with 5% CO_2_ at 37 °C, and the culture medium was changed on alternate days. When 70–75% confluent, the cells were detached using ethylenediaminetetraacetic acid (EDTA, Invitrogen) and split in a 1:5 ratio. For blebbing, these F11 cells with a density of 20000 cells/mL were seeded in a T25 culture flask and left in the incubator for at least 72 h. Production of cell-free GPMVs was carried out as per published protocol^13,14^ using vesiculation buffer containing *N*-ethylmaleimide (NEM) at a concentration of 2 mM in GPMV buffer, which was a solution made up of 2 mM CaCl_2_·2H_2_O, 10 mM 4-(2-hydroxyethyl)piperazine-1-ethane-sulfonic acid (HEPES), and 0.15 M NaCl, each in deionized (DI) water, adjusted to a pH of 7.4 using 1 M NaOH (all from Sigma-Aldrich). Briefly, cell media were poured out from the culture flasks, and the adherent cells were rinsed twice with 2 mL of GPMV buffer. Then 2 mL of GPMV+NEM buffer was added, and the flasks were placed inside a rocking incubator (Zhicheng) set to 37 °C and 50 rpm for 1 h. Supernatant was collected from each flask into a single falcon tube placed vertically on a thermoshaker (IKA) at 1 °C for 1 h. Cell debris that settled as a pellet at the bottom of this tube was then carefully removed, leaving behind F11 blebs in the GPMV+NEM vesiculation buffer.

### Synthetic Vesicle Extrusion

DOTAP vesicle solution (1 mg/mL in PBS) was prepared as per previous work^8^ using a polycarbonate membrane with 100 nm pores (Whatman Nuclepore) in a mini extruder kit (Avanti), to serve as the reference sample for DLS measurements.

### DLS

A Zetasizer Nano ZS90 (Malvern Panalytical) with a 4 mW 632.8 nm laser light source was used to obtain the particle size distribution and zeta potential of F11 blebs in GPMV+NEM buffer as well as DOTAP vesicles (0.1 mg/mL), ETE-S (0.1 mg/mL), and HRP (1 mg/mL) in PBS buffer. Disposable UV-transparent polystyrene cuvettes with caps (Sarstedt) containing 100 μL (for size) or 1 mL (for zeta) of the sample solution were loaded into the instrument, and measurement was conducted by selecting material as lipid or protein, dispersant as water, equilibration time of 60 s, and scattering detector angle of 90° in the Zetasizer Software v. 8.02. Average data were obtained from three measurements per sample with at least 10 runs of 10 s each per measurement and processed using the general purpose analysis model.

### EQCM-D

A QSense E4 Analyzer (Biolin Scientific) and high-precision multichannel IPC pump (ISMATEC) were used to measure frequency and dissipation values related to material adsorption on Au coated quartz sensors (5 MHz, Biolin Scientific) mounted inside regular flow modules. Simultaneous QCM-D and electrochemistry was also conducted by connecting a μAutolab III potentiostat (Metrohm) to the QCM-D setup, where the 3-electrode electrochemical system consisted of a Au–Ti coated quartz sensor (5 MHz, Biolin Scientific) as the working electrode, an external Dri-ref Ag/AgCl (World Precision Instruments) as the reference electrode, and the Pt plate (Biolin Scientific) of the EQCM-D module as the counter electrode. QCM-D sensors were immersed in TL1 cleaning solution (DI water, 31% H_2_O_2_, and 25% NH_4_OH mixed in a ratio of 5:1:1) at 100 °C for 7 min, rinsed using DI water, dried using N_2_ stream, and placed in an ultraviolet (UV)-ozone cleaner (Novascan Technologies) for 30 min to further decontaminate and render the surface hydrophilic. Sample solutions were introduced into the QCM-D modules at 0.1 mL/min flow rate after establishing a stable baseline in PBS for at least 15 min prior to each measurement. Electrochemical impedance spectroscopy (EIS) was measured during the PBS rinse stage in the EQCM-D module at 0 V against the open-circuit potential by applying a 10 mV AC sinewave input signal whose frequency was scanned from 1 MHz to 0.1 Hz at a distribution of 10 points per decade. Resulting data were acquired and analyzed using QSoft, QTools, QSense Dfind 1.2.7, Nova 2.1, and Origin 2024 softwares.

### Peroxidase Assay

Activity of the HRP enzyme adhering to the F11 bilayer was examined on the lipid-bilayer-modified QCM-D sensors according to the standard procedure using the Enhanced K-Blue TMB substrate.^8,16,17^ After the introduction of 0.5 mL of F11 blebs in GPMV+NEM buffer solution and the corresponding PBS rinse, 1 mL of HRP solution at 1 mg/mL in PBS was introduced into the same QCM-D flow module, followed by a PBS rinse. Three such sensors were removed from their respective modules while still wet and quickly but carefully immersed in vials containing 0.5 mL of PBS each, to which 0.5 mL of the chromogenic TMB substrate solution was added, and the samples were covered with a lid and set aside to incubate for 30 min under ambient conditions. Absorbance data were then obtained at a wavelength of 450 nm using a Synergy H1 microplate reader (Bio-Tek) by transferring 50 μL of each of the vial contents into a well of a 96-well F-bottom clear polypropylene microplate (Greiner Bio-One) having 50 μL of a 1 N H_2_SO_4_ solution to stop the enzyme–substrate reactions after 5 min incubation outside the instrument, and 1 min orbital shake inside the instrument. A reference solution well containing 25 μL of PBS, 25 μL of TMB, and 50 μL of 1 N H_2_SO_4_ to match the mix and volume level of sample solution wells was measured and subtracted from the sample absorbance values.

### Spectroscopy

The microplate reader setup described in the previous section was also used to observe *ex situ* polymerization of ETE-S in the presence or absence of F11 blebs and HRP enzyme solutions. Equal volumes of the inflow samples used for QCM-D measurements, i.e., 25 μL each of F11 blebs, HRP at 1 mg/mL, and ETE-S at 0.1 mg/mL with H_2_O_2_ at 1 mM, were directly mixed as required in different wells of the 96-well microplate; 50 μL volumes of individual sample or buffer solutions were also used for reference. The microplate was covered with its lid (both transparent) and left aside to incubate for 15 min under ambient conditions, after which the excess 25 μL was removed from particular wells to ensure common total volume of 50 μL in each well of the microplate. Average absorbance spectra in the 300–990 nm range were then measured for each sample (3 scans per well, 10 nm/step), after 1 min orbital shake.

### AFM

A ScanAsyst-Fluid cantilever fitted to a Dimension Icon XR (Bruker) instrument operating in ScanAsyst-Fluid mode was used to observe the Au/Ti quartz sensors after EQCM-D measurement, while still covered with PBS. Nanoscope v10 software was used to acquire the images from the central region of the sensors (5 μm^2^ scan size), and Gwyddion 2.65 was used to analyze them after basic operations like correcting horizontal scars, aligning rows using the median method, leveling data by mean plane subtraction, and shifting the minimum data value to zero.

### Confocal Microscopy and FRAP

Some of the F11 bleb samples used for QCM-D measurements were fluorescently labeled by adding octadecyl rhodamine B chloride (R18) dye (Invitrogen) at 2 μg/mL to the blebs in GPMV+NEM buffer solution at room temperature and gently shaking the container by hand to stain the blebs without damaging them. After the QCM-D measurement to form the bleb bilayer, the Au quartz sensor was removed from the module while still wet and carefully placed upside down in 300 μL of PBS enclosed within a polydimethylsiloxane (PDMS) well on a clean glass coverslip (VWR) and imaged using the 20× objective of an inverted Zeiss LSM 980 confocal microscope (Carl Zeiss) with a 561 nm laser and Rhodamine B fluorescence channel. FRAP measurements were also done on this sensor by bleaching three 20 μm diameter circular regions for ∼6 min and observing the recovery of fluorescence intensity in these bleached regions relative to a 30 μm diameter reference spot for ∼16 min. Images were acquired using ZEN 3.4 and processed using Microsoft Photos; FRAP data were analyzed using EasyFRAP-web 1.12 application.^18^

## Results and Discussion

Real-time QCM-D was employed to characterize the adsorption processes upon the sequential introduction of F11 blebs, HRP, and ETE-S with H_2_O_2_ on a Au/Ti quartz sensor. ETE-S is enzymatically polymerized by the adsorbed HRP to form PETE-S as depicted in Figure 1. The enzyme HRP has a broad selectivity for substrates and has been shown to polymerize ETE-S with H_2_O_2_ acting as the oxidant.^4,19−21^ A PBS buffer rinse was performed after each sample adsorption stage, at which point the complementary EIS measurement was recorded. The surface-confined processes in the absence of HRP and H_2_O_2_ were also evaluated, as control measurements. Entire QCM-D measurements spanning F11 bleb bilayer formation, HRP adsorption, ETE-S adsorption, and polymerization stages are shown in Figure S1, while the corresponding data for the individual stages are presented in the subsequent sections. QCM-D frequency shifts are proportional to the film’s hydrated mass changes, while dissipation shifts reflect alterations in the film’s viscoelastic properties. A frequency drop (i.e., a more negative frequency compared to the reference point at the start of the measurement) is associated with an increased adsorbed mass, whereas a dissipation rise (i.e., a more positive dissipation) is linked to a increased softness of the film. Also, the distribution of overtones indicates these changes across the material bulk, with the lower overtones representing changes in the top-most surface of the adsorbed material.^8,22^

### Bleb Bilayer Formation

**Figure 2:**
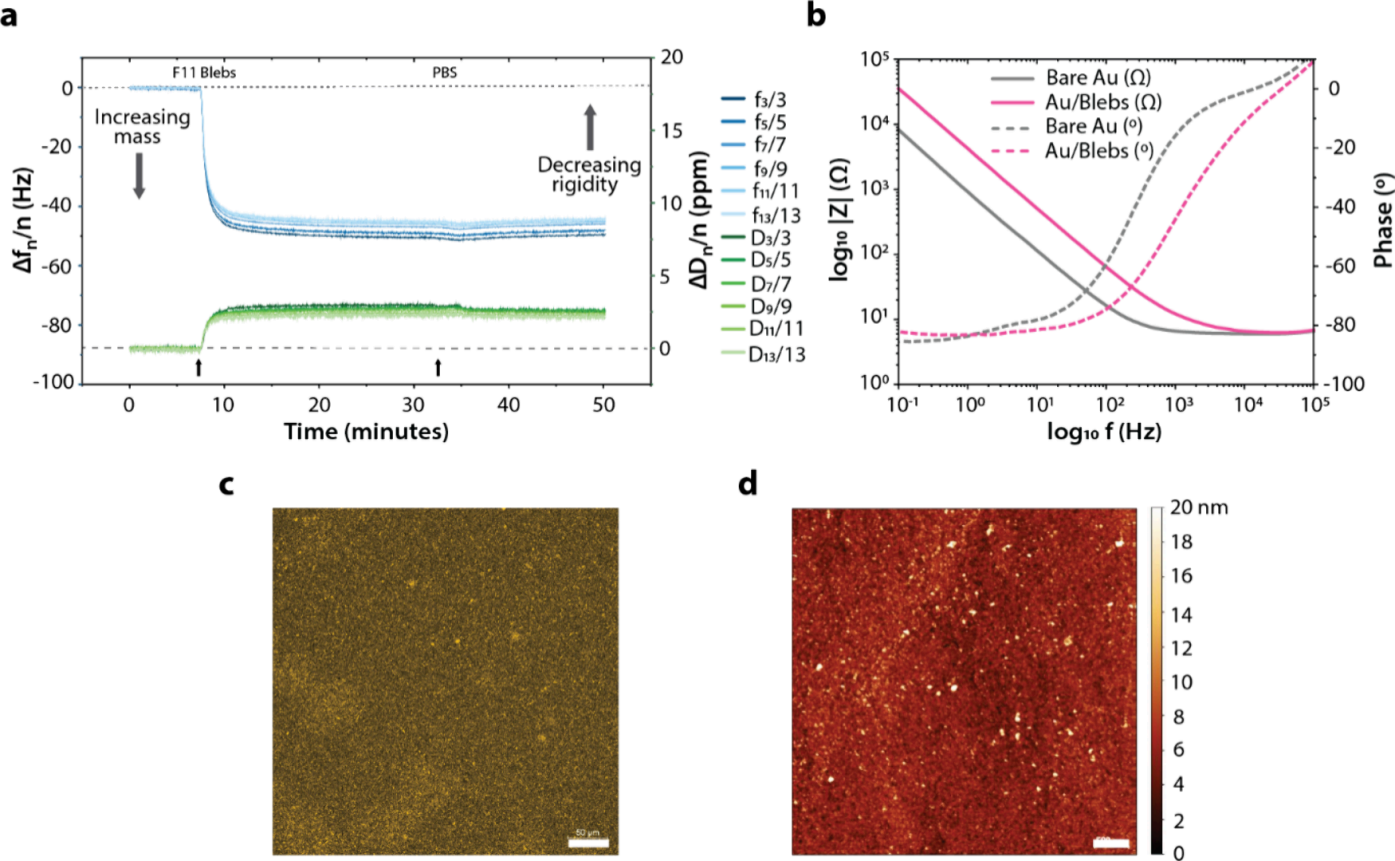
F11 blebs adhesion and bilayer formation on Au: (a) Overtone-normalized *Δf* and *ΔD* plots for all measured overtones (*n* = 1, 3, 5, ..., 13) and (b) EIS signals related to the formation of an F11 bleb bilayer on a Au surface, along with the *ex situ* (c) confocal microscopy (scale bar 50 μm, λ_ex_/λ_em_ 556 nm/578 nm) and (d) AFM (scan size 5 μm^2^, scale bar 500 nm, surface roughness 1.85 nm) images taken near the central region of the bleb-bilayer modified QCM-D sensor in PBS.

Introduction of 0.5 mL of F11 blebs in GPMV+NEM buffer into the QCM-D module caused immediate adsorption and rupture of the blebs on the Au surface to create a bleb bilayer, as seen in Figure 2a (black arrows near the horizontal axis indicate the time when solutions were introduced). Corresponding EIS spectra measured during the respective PBS rinse stage are shown in Figure 2b.

Frequency and dissipation shifts are fairly consistent across batches (*Δf*_7_ −47.0 ± 1.0 Hz and *ΔD*_7_ 2.7 ± 0.3 ppm, mean ± standard error, *n* = 3), and similar to the reported frequency and dissipation shifts of −50 Hz and 5 ppm respectively confirming SLB formation for HeLa-cell based blebs on SiO_2_ surfaces.^9^ However, corresponding EIS signals show larger than expected increases in impedance for the bleb bilayer, probably owing to the presence of membrane debris left over from the vesiculation process (Figures S4–S6) along with the mostly smooth and tightly packed lipid bilayer seen in Figures 2c and 2d. This could indicate that the bleb bilayer may not be present as a flat or completely uniform layer across the entire sensor surface.

### HRP Adsorption

**Figure 3:**
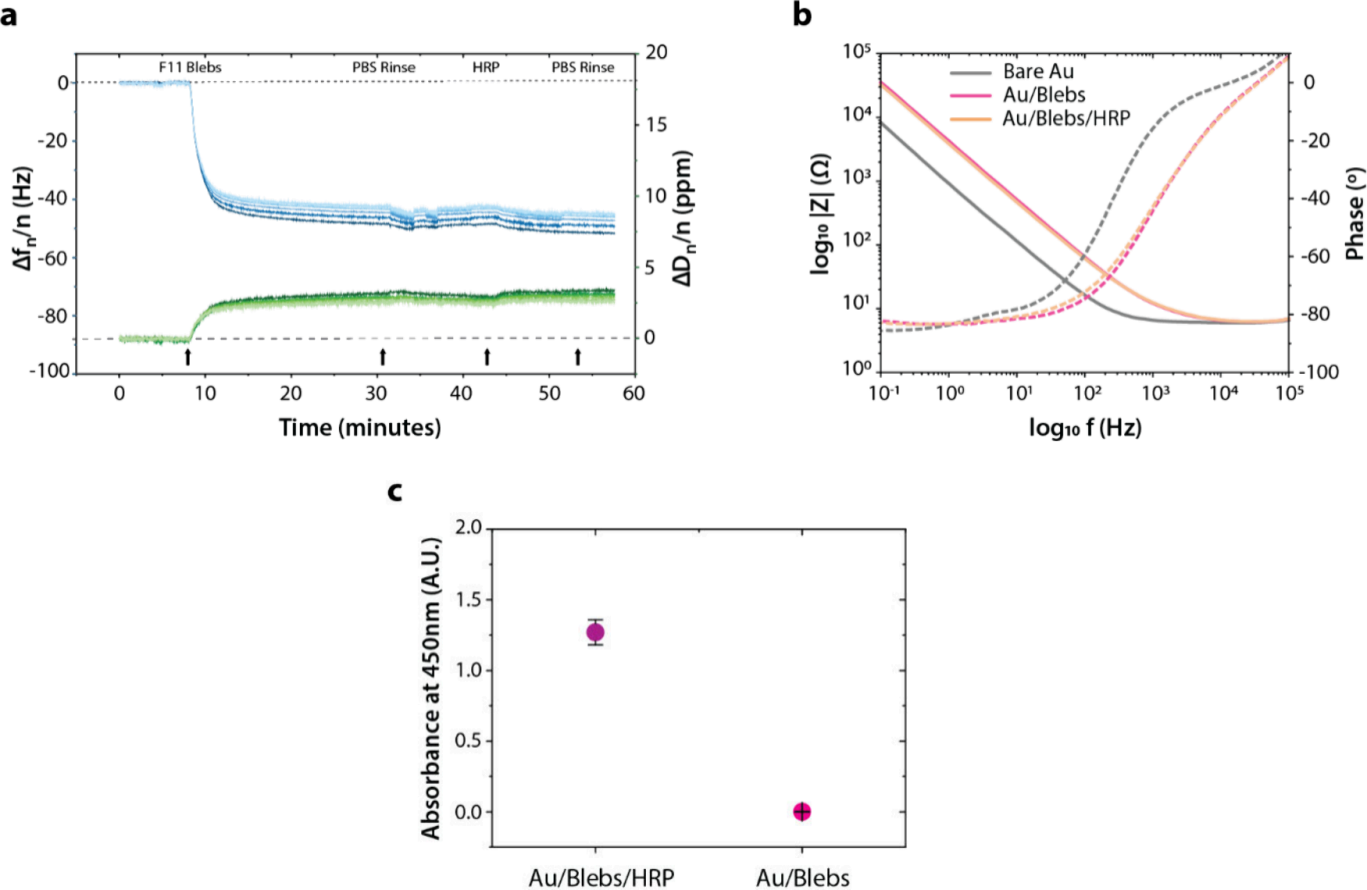
HRP adhesion on Au/Blebs and peroxidase activity assay: (a) Overtone-normalized *Δf* and *ΔD* plots for all measured overtones (*n* = 1, 3, 5, ..., 13) and (b) EIS signals related to the adsorption of HRP on the F11 bleb bilayer supported on a Au surface (bare Au and Au/Blebs repeated from Figure 2b for comparison), along with the (c) results of the *ex situ* TMB assay indicating peroxidase activity in native and HRP-adsorbed SLB membrane on the Au/Ti quartz sensors; error bars represent standard error, *n* = 3.

Introduction of 1 mg/mL HRP enzyme in PBS caused minimal shift in frequencies and dissipation or in the impedance of the system as seen in Figures 3a and 3b, possibly indicating that HRP does not cover the bleb bilayer completely, couples to flexible components extending from the membrane, or integrates into liquid regions replacing water, or some combination of these mechanisms. However, whatever does adhere seems to be quite active, consistently across different trials, as indicated in the results from a peroxidase activity assay (TMB Assay, Figure 3c) conducted on samples collected from surface-modified sensors immersed in PBS after QCM-D measurements (after 30 min incubation of the sensors with TMB).

### ETE-S Adsorption and Polymerization

**Figure 4:**
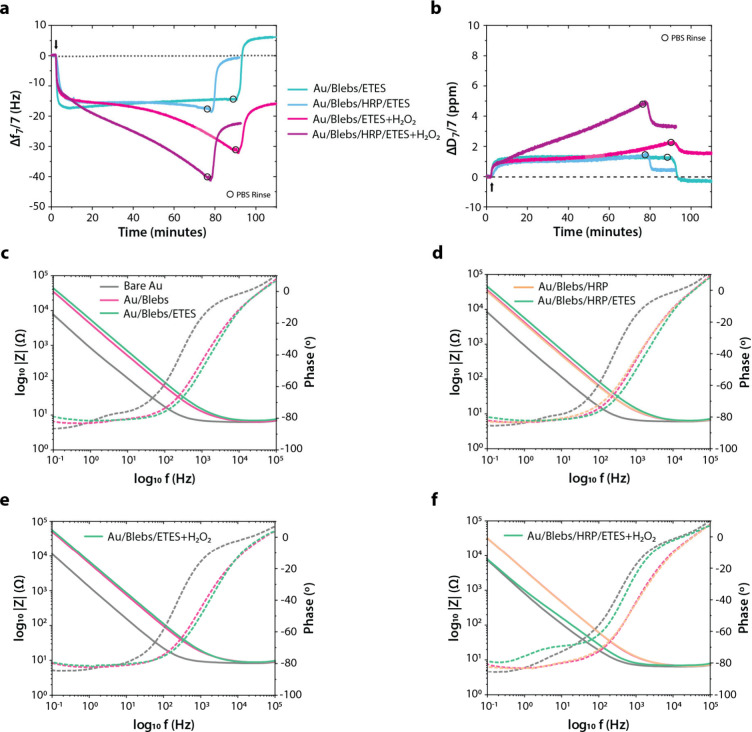
Comparative representation of ETE-S introduction and polymerization: Seventh-overtone-normalized (a) frequency and (b) dissipation shifts over time during adsorption and enzymatic polymerization of ETE-S on native and HRP-modified F11 native SLB membrane on a Au surface, relative to the previous layer as a reference (arrows indicate the time instant when ETE-S was introduced with or without H_2_O_2_, and circles indicate the time instant when PBS rinse was performed), along with corresponding (c–f) EIS Bode plots for the four systems.

Enzymatic polymerization of ETE-S monomer adsorbed on the F11 bleb SLB surface, both in the presence and absence of HRP, is shown in Figure S1. A solution containing ETE-S and H_2_O_2_ at respectively 0.1 mg/mL and 1 mM in PBS was introduced into the QCM-D flow module to detect possible enzymatic polymerization on both the native and HRP-modified F11 bleb bilayer. To evaluate the effects of monomer interaction with the bilayer surface and distinguish them from the effects of polymerization, ETE-S adsorption in the absence of H_2_O_2_ was also tracked separately using EQCM-D. The frequency and dissipation responses were recorded continuously for at least 1 h under no-flow condition, after which a PBS rinse was performed, and EIS was measured to determine polymer formation by the reduction of overall electrode impedance owing to the increase in the electroactive surface area or volume. A comparative view of the frequency and dissipation shifts corresponding to the seventh overtone during this stage of the measurement is presented in Figures 4a and 4b, respectively. The seventh overtone, which was regularly observed as a relatively clean signal, was chosen as a characteristic of the data. The corresponding Bode plots indicating the impedance and phase changes of the four systems are presented in Figures 4c–4f.

Although ETE-S seemed to initially adsorb at a similar rate (overlapping data near *t* = 0 min in Figures 4a and 4b), polymerization seemed to occur only when H_2_O_2_ was present, as indicated by the larger *f*_7_ and *D*_7_ shifts in the pink and purple plots of Figures 4a and 4b, compared to the blue and green plots. The final PBS rinse (peaks or steps in the 75–95 min range) removed water-soluble monomer portions that poorly attached to the bleb bilayer, while the water-insoluble polymer portions remained attached to the F11 SLB. EIS data show impedances (green) higher than those corresponding to blebs (red) or HRP (orange) when H_2_O_2_ was absent (Figures 4c and 4d), but comparatively lower and almost overlapping the blebs or bare Au responses (gray) when H_2_O_2_ was present (Figures 4e and 4f). Particularly in Figure 4f, a significant decrease in the impedance related to PETE-S, which is lower than that of Au/Blebs/HRP at low frequencies, could be due to the polymer making contact with the underlying Au electrode surface through possible gaps in the bleb bilayer. It is interesting to observe mass increase even in the absence of HRP (pink plot in Figures 4a and 4b), suggesting either monomer aggregation or PETE-S formation perhaps due to polymerization from native enzymes present in the F11 bleb bilayer. Although corresponding EIS data (green plot in Figure 4e) would indicate much higher impedance and much farther right-shifted phase signals compared to that of the bleb bilayer (pink plot in Figure 4e), if this mass increase was due to the formation of an insulating layer, further experiments and analyses are required to conclusively determine the nature and cause of this result. Frequency and dissipation shifts follow a similar trend for these four systems across multiple trials, as evidenced by the final *f*_7_ and *D*_7_ values presented in Figure S2. Shifts in *f*_7_ and *D*_7_ beyond the reference level for the case of ETE-S on the bleb bilayer and significant impedance drop for the case of PETE-S on the HRP-modified bleb bilayer could indicate that ETE-S breaches the bilayer and directly contacts the Au substrate underneath. However, the corresponding frequency and dissipation shifts along with the EIS plots suggest that the bleb bilayer is not disrupted in this case, unlike in previous work involving ETE-S polymerization on the DOTAP bilayer,^8^ where the synthetic lipid bilayer was disrupted owing to the stronger electrostatic interactions between oppositely charged cationic DOTAP lipids and anionic PETE-S.

### Polymer Morphology

**Figure 5:**
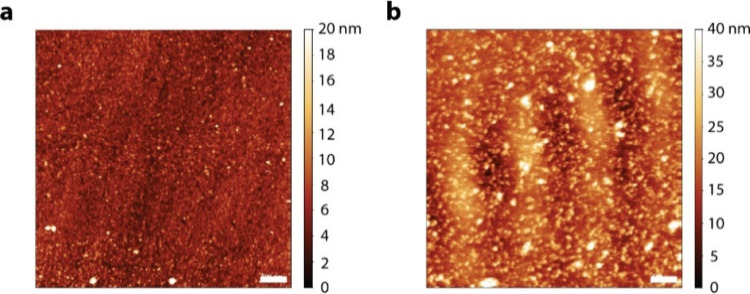
AFM images for (a) Au/Blebs/ETES+H_2_O_2_ and (b) Au/Blebs/HRP/ETES+H_2_O_2_, taken near the central region of the Au/Ti quartz sensors in PBS; 5 μm^2^ scan size and 500 nm scale bar.

The morphology and the topography of the enzymatically polymerized PETE-S film adhering to the native or HRP-modified bleb bilayer (supported on the Au substrate and still covered in PBS) were investigated using AFM and are presented in Figure 5.

Surface roughness values are 1.87 nm for Au/Blebs/ETES+H_2_O_2_ (without HRP, Figure 5a) and 5.55 nm for Au/Blebs/HRP/ETES+H_2_O_2_ (Figure 5b). The surface topography appears even for each image. The roughness value for Figure 5a is almost the same as that of the F11 bleb bilayer in Figure 2d, possibly indicating the presence of a very thin layer or of small aggregates. However, no disruption to the bilayer seems to be apparent in either case.

To summarize, ETE-S was enzymatically polymerized by HRP on the F11 bilayer membrane only when H_2_O_2_ was present as the oxidant for the reaction. EQCM-D results (Figure 4) indicate a possible breach of the bleb bilayer. However, the AFM data (Figure 5) do not show any disruption to the bilayer upon polymerization, suggesting that ETE-S could be penetrating through gaps or defects in the bilayer. The mass increase upon introduction of ETE-S and H_2_O_2_ in the absence of HRP suggests either monomer aggregation or PETE-S formation perhaps due to polymerization from native enzymes present in the F11 bleb bilayer. Published research shows that blebs rupture through a “parachute” mechanism on the supporting substrate, whereby the outer leaflet of the spherical GPMVs in solution becomes the top leaflet of the planar SLB.^9,23,24^ PETE-S formation in the absence of HRP would therefore be attributed to the activity of some native enzymes present in the bottom leaflet of the F11 bilayer, considering that polymerization is observed in the sensitive EQCM-D system where F11 blebs have ruptured to form a bilayer, but not in the microplate reader instrument where the F11 blebs are still in their unruptured state (Figures S3 and S8). The exact identity of this enzyme or group of enzymes is beyond the scope of current work, although TMB assay results (Figure 3) rule out native cell membrane peroxidases exposed on the top leaflet of the bilayer from being responsible for these polymerization reactions. Regardless, addition of HRP catalyzes the enzymatic polymerization of ETE-S, resulting in higher gravimetric capacitance and lower Young’s modulus values of the resulting polymer, compared to that of the material formed in its absence (Figure S6 and Tables S1 and S3). Faster rates of polymerization and larger amounts of deposited material are also observed for the HRP-modified bleb bilayer compared to the native bleb bilayer, when similar QCM-D measurements are carried out at a higher temperature of 37 °C, owing to temperature-dependent reaction kinetics as well as increased enzyme activity (Figure S7).

## Conclusion

Simultaneously measured microgravimetric and impedance data are used to showcase the feasibility of *in situ* enzymatic polymerization of ETE-S on a biomimetic native membrane derived from mammalian cells, both at room temperature of 22 °C and mammalian body temperature of 37 °C. Water-soluble monomer polymerizes in the presence of H_2_O_2_, due to the activity of externally introduced HRP on the F11 bleb bilayer, to form a polymer film that adheres to the underlying substrate. Although the bleb bilayer model is not identical to whole cells despite being a good research substitute, the activity of native enzymes cannot be ignored when considering enzymatic polymerization of ETE-S and similar materials in live cells and tissues or in future animal studies for potential *in vivo* development of next-generation neural electrodes.

## Supporting Information Available

QCM-D recordings of the four samples for the entire measurement duration, modeled parameters from QCM-D data of the samples for all measured overtones, seventh-overtone-normalized frequency and dissipation shifts at the end of measurements, DLS distributions along with zeta potential values for samples in solution, FRAP images and analysis, comparative seventh-overtone-normalized frequency and dissipation plots for F11 blebs at three different concentrations along with corresponding *ex situ* confocal microscopy images of the QCM-D sensors, impedance and phase spectra of various EQCM-D measurements, values of the fitted circuit elements as well as gravimetric capacitance of the polymers obtained using modeling software, comparative seventh-overtone-normalized frequency and dissipation plots for *in situ* polymerization of PETE-S on Au/Blebs and Au/Blebs/HRP along with TMB assay results indicating HRP and native peroxidase activities at room and body temperatures, and absorbance spectra for all the QCM-D measurement samples (PDF)
