# Supplementary material for: Enzymatically Polymerized Organic Conductors on Native Lipid Membranes
Source: Langmuir. 2024 Dec 17;40(52):27299–306. doi: 10.1021/acs.langmuir.4c03373 (PMC11697344; doi:10.1021/acs.langmuir.4c03373)
Supplement: Supplementary file 1 — la4c03373_si_001.pdf [file la4c03373_si_001.pdf]

SUPPORTING INFORMATION

Enzymatically polymerised organic conductors on native lipid membranes

Diana Priyadarshini[1], Tobias Abrahamsson[1], Hanne Biesmans[1], Xenofon Strakosas[1], Jennifer Y. Gerasimov[1], Magnus Berggren[1], Daniel T. Simon[1], and Chiara Musumeci[1]*

1 Laboratory of Organic Electronics, Department of Science and Technology, Linköping University, 60174 Norrköping, Sweden

* Corresponding author: chiara.musumeci@liu.se

- QCM-D recordings of the four samples for the entire measurement duration,
- Modelled parameters from QCM-D data of the samples for all measured overtones,
- Seventh overtone-normalised frequency and dissipation shifts at the end of measurements,
- DLS distributions along with zeta potential values for samples in solution,
- FRAP images and analysis,
- Comparative seventh overtone-normalised frequency and dissipation plots for F11 blebs at three different concentrations along with corresponding *ex situ* confocal microscopy images of the QCM-D sensors,
- Impedance and phase spectra of various EQCM-D measurements,
- Values of the fitted circuit elements as well as gravimetric capacitance of the polymers obtained using modelling software,
- Comparative seventh overtone-normalised frequency and dissipation plots for *in situ* polymerisation of PETE-S on Au/Blebs and Au/Blebs/HRP along with TMB assay results indicating HRP and native peroxidase activities at room and body temperatures, and
- Absorbance spectra for all the QCM-D measurement samples

Number of Pages: 10

Number of Figures: 8

Number of Tables: 3

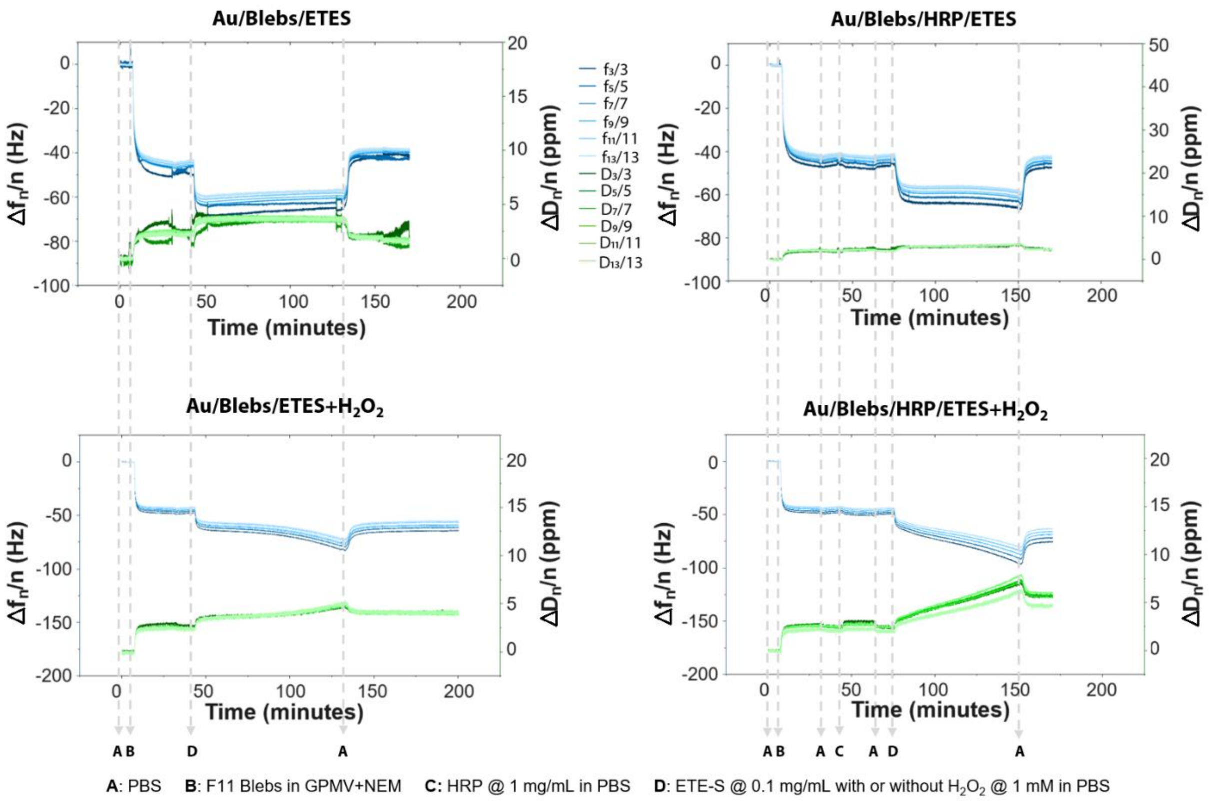

Figure S1. Overtone-normalised QCM-D recordings of the four samples for the entire measurement duration. Overtone spreading indicates a thick film, while energy dissipation values close to zero indicate a rigid film[1–3].

Table S1. Modelled parameters at the end of QCM-D measurement of the polymer samples, using Kelvin-Voigt viscoelastic model ('broadfit') relative to previous PBS rinse stage as reference and considering all measured overtones; mean ± standard error, n = 3.

Sample	Thickness (nm)	Specific mass (μg/cm^2)	Viscosity (mPa·s)	Shear modulus (KPa)
Au/Blebs/ETES+H2O2	25.4 ± 12.7	2.5 ± 1.2	1.5 ± 0.4	248.9 ± 245.5
Au/Blebs/HRP/ETES+H2O2	52.1 ± 2.5	5.1 ± 0.2	2.3 ± 1.2	89.8 ± 83.6

Comparatively higher error rates for shear (or elastic) modulus[4] and viscosity values are probably due to their relatively higher sensitivity on the statistical deviation in the measured frequency and dissipation shifts caused by their coupled dependence on these parameters, as opposed to the linear dependence of Δf and ΔD on the modelled specific mass and thickness values[1,5]. Shear moduli of the polymers are converted to Young's moduli by multiplying corresponding values by 2.7, with the assumptions of the material being isotropic and having a Poisson ratio of 0.4[6,7]. The resulting average Young's moduli of 0.2 MPa and 0.7 MPa for the material on Au/Blebs/HRP and Au/Blebs substrates, respectively, are lower

than the reported 10 MPa to 3 GPa range of other solid or thin-film conjugated polymers and closer to the ~0.01 MPa of biotic living tissue and ~0.6 MPa of human skin[1,8,9].

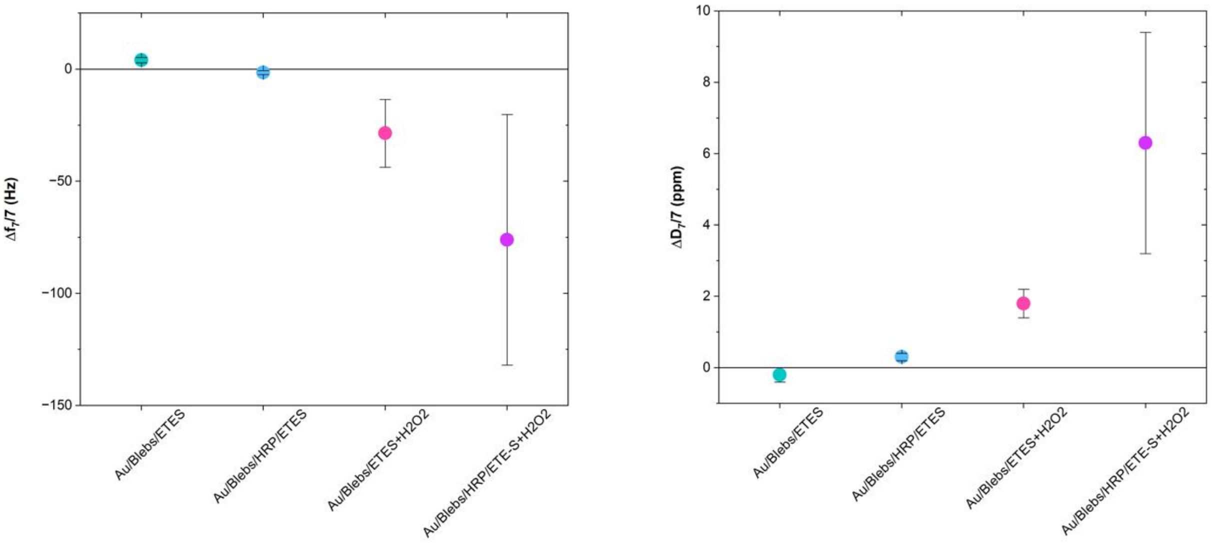

Figure S2. Seventh overtone-normalised frequency and dissipation values at the end of corresponding QCM-D measurement, relative to previous PBS rinse stage as reference; error bars represent standard error, n = 3.

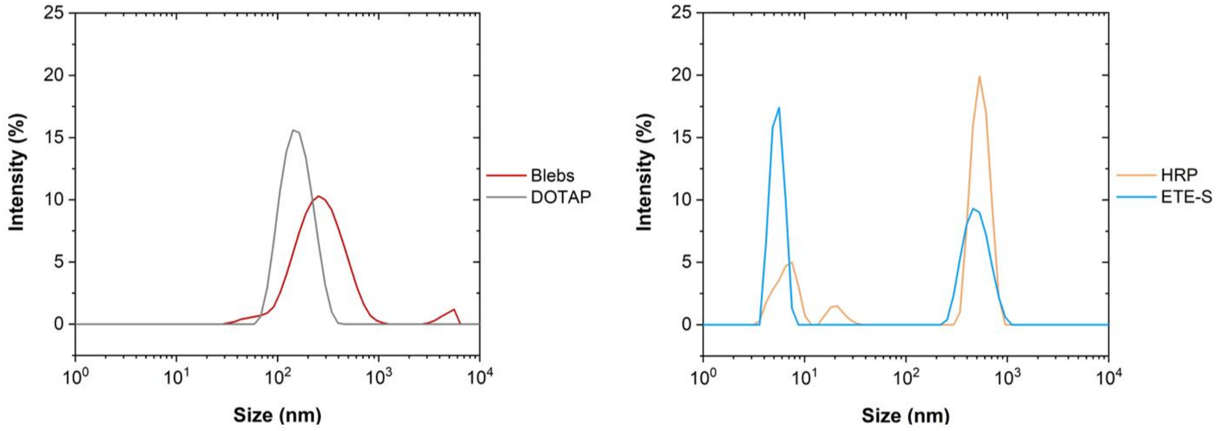

Figure S3. Dynamic light scattering (DLS) measurement of average size distribution of particles suspended in GPMV+NEM (blebs) or PBS (DOTAP, HRP, ETE-S) buffers; prominent single peak indicates the presence of spherical particles, while multi-peak distribution indicates the presence of different sized aggregates; slightly larger size of 100 nm extruded DOTAP vesicles is due to the DLS instrument considering hydrodynamic radius of the suspended particles; small peak closer to the micron

range for F11 blebs could be due to cell debris left over from the chemically induced vesiculation process.

Table S2. Zeta potentials for the particles suspended in buffer; mean ± standard error, n = 3.

Sample	Zeta Potential (mV)
F11 Blebs in 7.4 pH GPMV+NEM	-11.4 ± 0.6
DOTAP at 0.1 mg/mL in PBS	43.4 ± 2.0
HRP at 1 mg/mL in PBS	-4.6 ± 0.6
ETE-S at 0.1 mg/mL in PBS	-45.4 ± 1.0

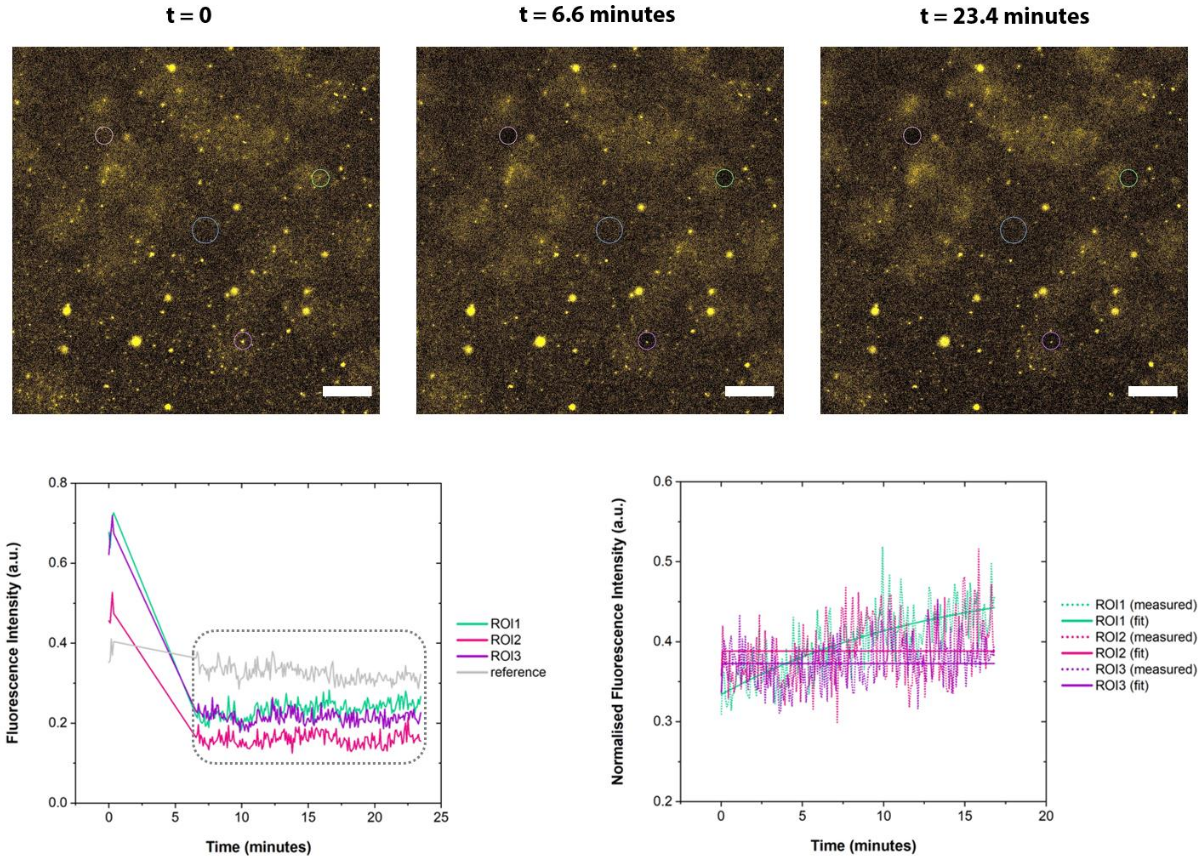

Figure S4. Confocal microscopy images of the F11 bleb bilayer-modified Au quartz sensor in PBS, taken at 0, 6.6, and 23.4 minutes of the FRAP measurement (scale bar 50 µm), along with quantitative fluorescence intensity plots of three bleached spots of 20 µm diameter relative to a single reference spot of 30 µm diameter; enhanced brightness, exposure, and contrast for the images performed using processing software; circuit fitting for the normalised intensities post-bleaching (highlighted with dotted rectangle) using the single exponential term equation $I_{fit} = I_0 - \alpha e^{-\beta t}$ where I_0 is the maximal intensity, while α and β are constants[10].

Good quality fit occurs for only one of the bleached spots (green), yielding the mobile fraction value of 0.49 and the half maximal recovery time τ_{half} of 9.5 minutes, from which the diffusion coefficient can be estimated as 0.044 μm²/s using the Soumpasis equation $D = r^2/(4\tau_{half})$ where r is the radius of the region of interest (10 μm in this case); these values are lower than reported values of bleb bilayers supported on glass or polymer substrates, indicating slower diffusion of lipids possibly due to the presence of cell debris, as well as the 'uncushioned' nature of the Au surface for the SLB[11,12] and blockages[13]. Additionally, size (hydrodynamic diameter) of the blebs in solution measured using DLS is in the range of 150 nm (Figure S3), whereas QCM-D modelling estimates the thickness of the bleb bilayer to be 20.0 ± 5.5 nm (mean ± standard error, n = 3), which could indicate the presence of a smooth bleb bilayer alongside patches of partially ruptured blebs or left-over cell debris (from the vesiculation process) on the Au sensor surface.

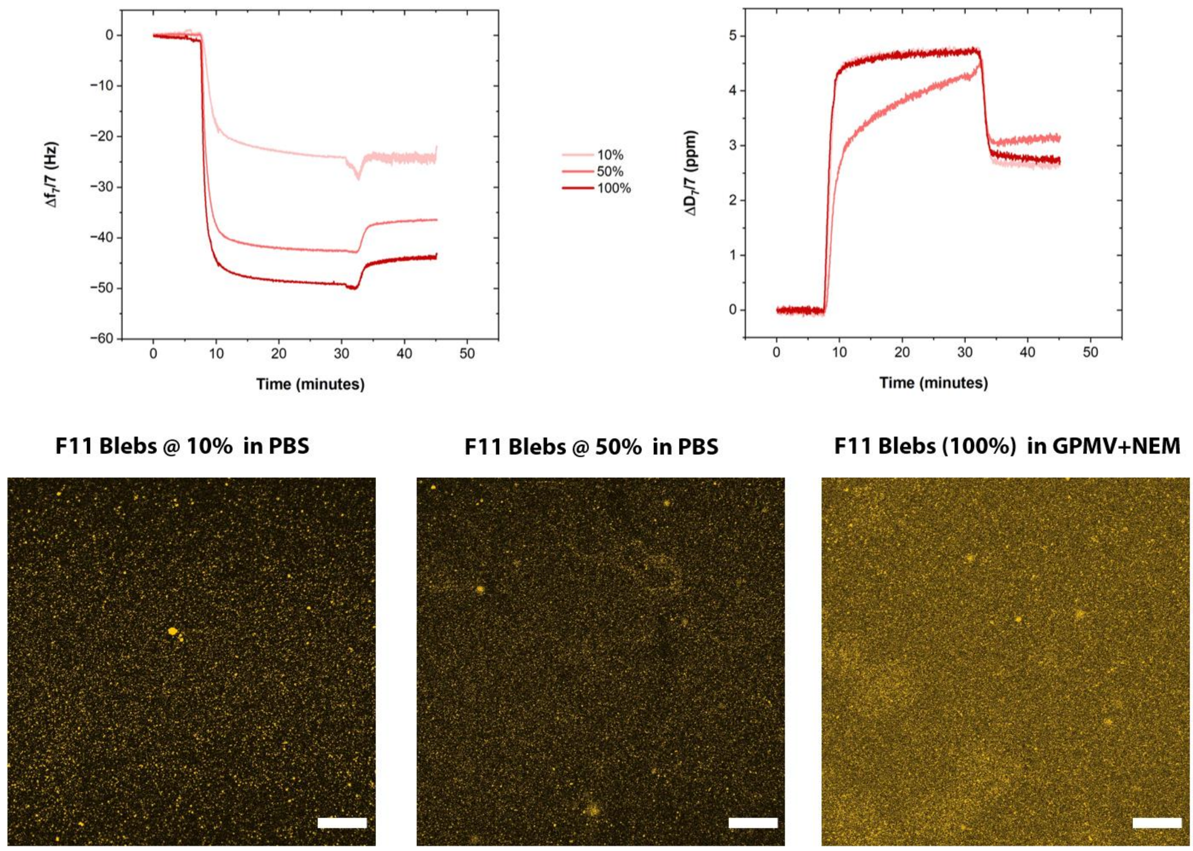

Figure S5. Seventh overtone-normalised frequency and dissipation plots for bleb layer formation at three dilutions of F11 blebs in PBS, along with corresponding confocal microscopy fluorescence images of the three Au quartz sensors in PBS after QCM-D measurement, indicating that the Au surface coverage is limited by solution concentration[14]; scale bars 50 μm; image for the 100% sample is the same as Figure 2c of main paper.

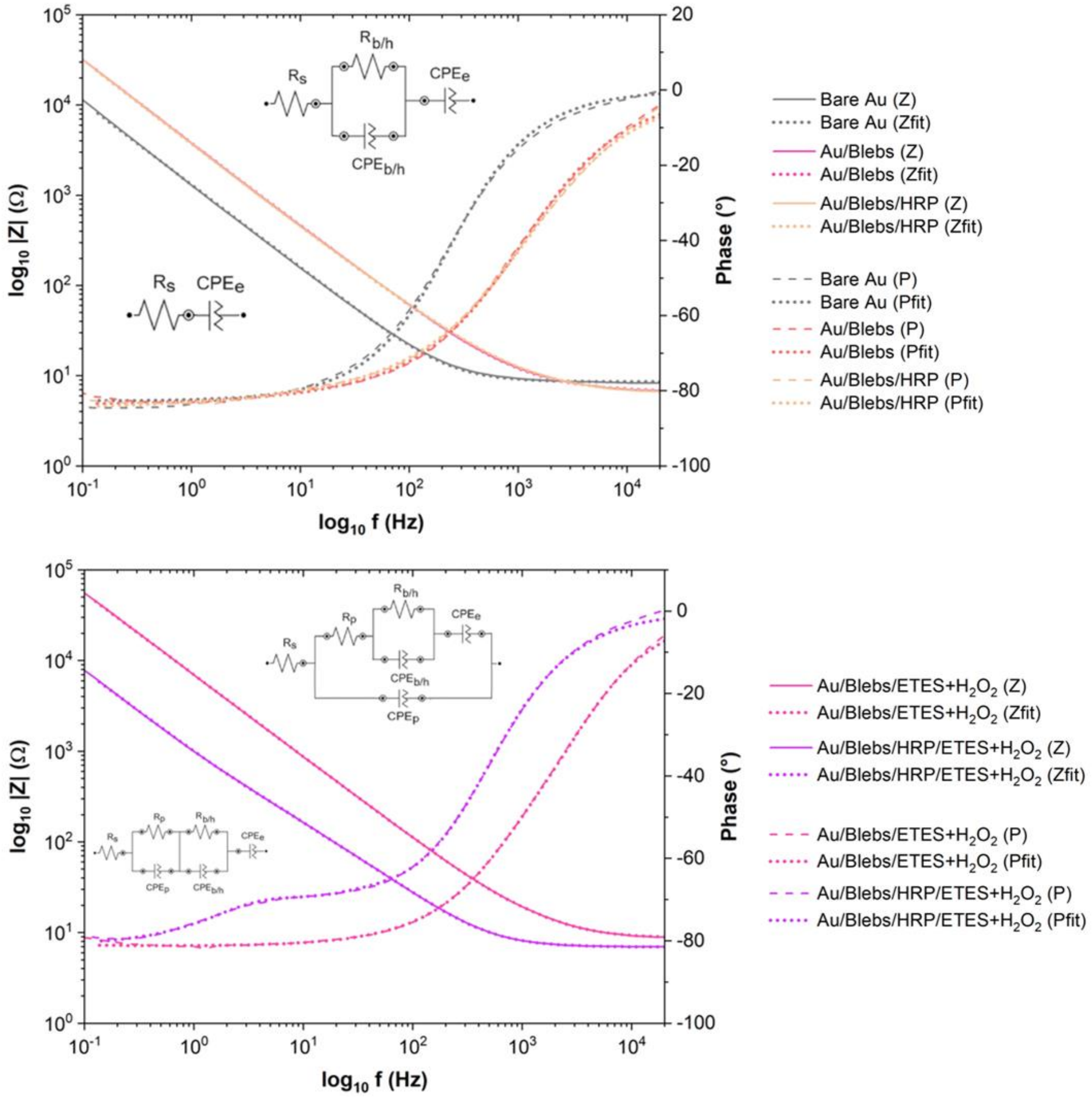

Figure S6. Impedance and phase spectra of various measurements where dotted plots represent the fit data modelled from the inset circuits, with the bottom-left circuit representing bare Au and the circuit on the top representing Au/Blebs or Au/Blebs/HRP or material grown *in situ* on Au/Blebs (bottom-left circuit) or Au/Blebs/HRP (circuit on top) substrates, considering that the material seems to breach the bleb bilayer to make contact with the underlying Au surface in the latter case but not in the former case; constant phase elements (CPEs) are used instead of an ideal capacitor for a better fit, considering the inhomogeneities of the layers and interfaces; capacitance is estimated using the equation $C = P\left({R_S}^{(1-\emptyset)}\right)^{(1-\emptyset)}$ where R_S is the solution resistance, P is the fitting parameter, and ϕ is an exponent factor with a value ranging from 0 for pure resistance to 1 for pure capacitance[15–17]; equivalent circuits

used for fitting of the EIS data include solution resistance R_s, electrode capacitance CPE_e, along with polymer resistance R_p and capacitance CPE_p, and bleb bilayer or HRP-modified bleb bilayer resistance and capacitance $R_{b/h}$ and $CPE_{b/h}$, respectively, similar to previous work[1].

Table S3. Values of the fitted circuit elements obtained using modelling software for frequencies ranging from 0.1 Hz to 20 KHz of the input AC signal; low ϕ values of several CPE elements indicate that resulting capacitances should be considered as mathematical estimates only[1,17].

Sample	R_s (Ω)	R_p (Ω)	CPE_p		C_p (μF)	$R_{b/h}$ (Ω)	$CPE_{b/h}$		$C_{b/h}$ (μF)	CPE_e		C_e (μF)
			$P_p \times 10^{-6}$ ($\Omega^{-1}s^{-\phi}$)	Φ_p			$P_{b/h} \times 10^{-6}$ ($\Omega^{-1}s^{-\phi}$)	$\Phi_{b/h}$		$P_e \times 10^{-6}$ ($\Omega^{-1}s^{-\phi}$)	Φ_e	
Bare Au	8.6	-	-	-	-	-	-	-	-	0.0	0.9	77.8
Au/Blebs	6.5	-	-	-	-	6590.3	0.0	0.6	86.8	0.0	0.9	28.1
Au/Blebs/HRP	6.3	-	-	-	-	8887.6	0.0	0.6	47.1	0.0	0.9	32.4
Au/Blebs/ETES+H2O2	7.0	130.4	0.0	0.9	51.1	371.6	0.0	0.7	8.6	0.0	0.9	41.0
Au/Blebs/HRP/ETES+H2O2	8.7	2.0	0.0	1.0	56.1	4736.3	0.0	0.8	113.6	0.0	0.9	13.0

Considering 5 mm diameter of the active sensing spot[18], gravimetric capacitances for PETE-S are calculated from capacitance values in this table and corresponding specific mass values in Table S1 as 41.4 F/g for polymer on Au/Blebs/HRP and 1.4 F/g for alleged polymer on Au/Blebs, which are in the range of reported values of PETE-S capacitances[1,19,20].

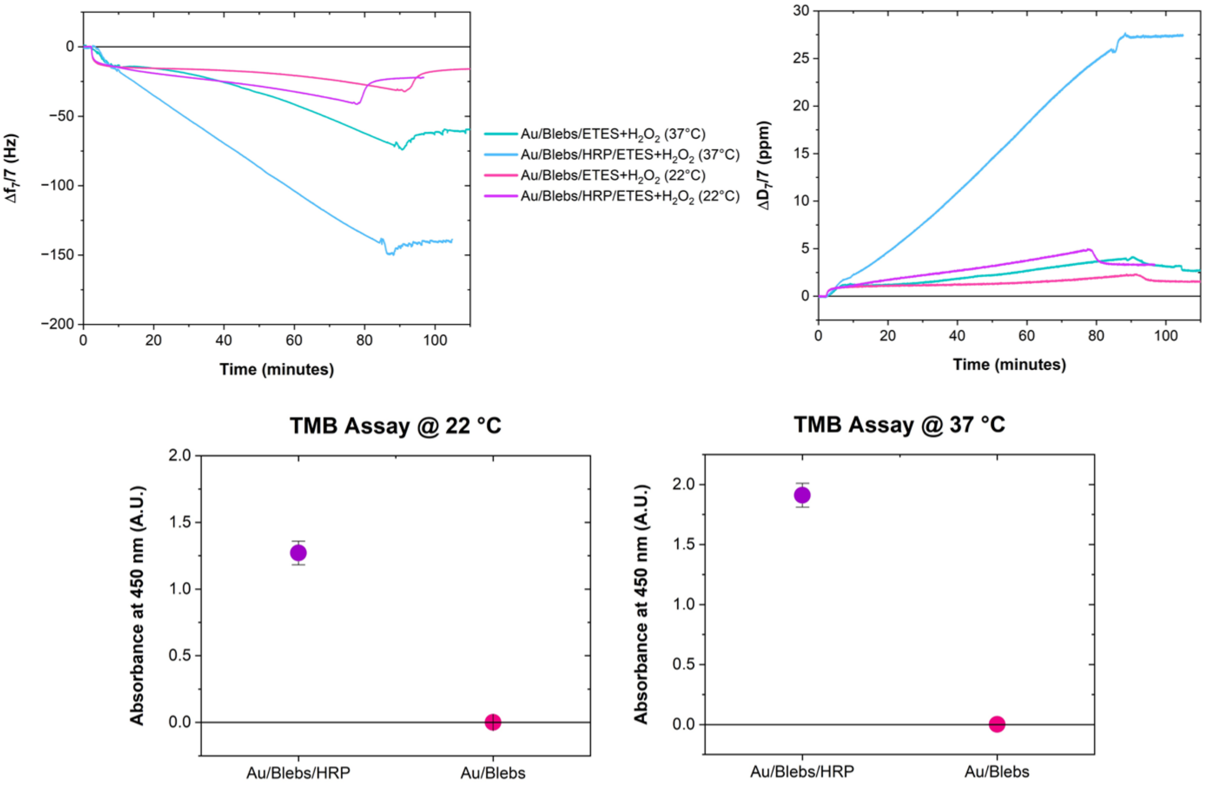

Figure S7. Overtone-normalised Δf_7 and ΔD_7 plots for Au/Blebs/ETES+H2O2 and Au/Blebs/HRP/ETES+H2O2, along with TMB assay results indicating peroxidase activity for these two

substrates; obtained from measurements conducted at temperature of 37 ºC, based on published temperature-based QCM-D studies.[21,22] Both frequency and dissipation shifts-follow a similar trend as at 22 ºC (plots same as those shown in Figures 4a and 4b of main paper), but deposition occurs at faster rates and in larger amounts, owing to temperature related kinetics as well as increased enzyme activity (TMB assay plot at 22 ºC is also same as Figure 3c of main paper).

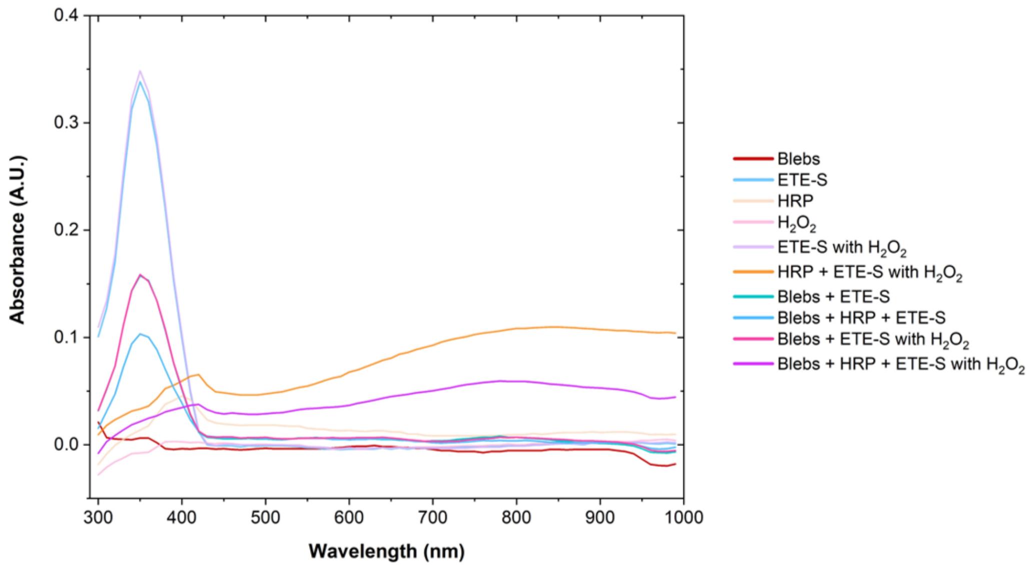

Figure S8. Blank-subtracted absorbance spectra for all the samples used in QCM-D recordings, measured using microplate reader, averaged from triplicates for each sample; monomer peak centred at 350 nm disappears when ETE-S gets enzymatically polymerised by HRP in the presence of H_2O_2 oxidiser, resulting in a broad peak starting at ~500 nm, confirming the doped state of PETE-S[20,23,24] (plate reader readings were taken after incubating the samples for 15 minutes).

References

(1) Priyadarshini, D.; Musumeci, C.; Bliman, D.; Abrahamsson, T.; Lindholm, C.; Vagin, M.; Strakosas, X.; Olsson, R.; Berggren, M.; Gerasimov, J. Y.; Simon, D. T. Enzymatically Polymerized Organic Conductors on Model Lipid Membranes. *Langmuir* **2023**, *39* (23), 8196–8204. https://doi.org/10.1021/ACS.LANGMUIR.3C00654.

(2) McCubbin, G. A.; Praporski, S.; Piantavigna, S.; Knappe, D.; Hoffmann, R.; Bowie, J. H.; Separovic, F.; Martin, L. L. QCM-D Fingerprinting of Membrane-Active Peptides. *Eur Biophys J* **2011**, *40* (4), 437–446. https://doi.org/10.1007/S00249-010-0652-5.

(3) Lu, N. Y.; Yang, K.; Li, J. L.; Yuan, B.; Ma, Y. Q. Vesicle Deposition and Subsequent Membrane–Melittin Interactions on Different Substrates: A QCM-D Experiment. *Biochim Biophys Acta - Biomembr* **2013**,

1828 (8), 1918–1925. https://doi.org/10.1016/J.BBAMEM.2013.04.013.

(4) McNamara, T. P.; Blanford, C. F. A Sensitivity Metric and Software to Guide the Analysis of Soft Films Measured by a Quartz Crystal Microbalance. *Analyst* **2016**, *141* (10), 2911–2919. https://doi.org/10.1039/C6AN00143B.

(5) Parveen, N.; Jana, P. K.; Schönhoff, M. Viscoelastic Properties of Polyelectrolyte Multilayers Swollen with Ionic Liquid Solutions. *Polym 2019, Vol 11, Page 1285* **2019**, *11* (8), 1285. https://doi.org/10.3390/POLYM11081285.

(6) Lubarsky, G. V.; Davidson, M. R.; Bradley, R. H. Hydration–Dehydration of Adsorbed Protein Films Studied by AFM and QCM-D. *Biosens Bioelectron* **2007**, *22* (7), 1275–1281. https://doi.org/10.1016/J.BIOS.2006.05.024.

(7) Root, S. E.; Savagatrup, S.; Pais, C. J.; Arya, G.; Lipomi, D. J. Predicting the Mechanical Properties of Organic Semiconductors Using Coarse-Grained Molecular Dynamics Simulations. *Macromolecules* **2016**, *49* (7), 2886–2894. https://doi.org/10.1021/ACS.MACROMOL.6B00204.

(8) Martin, D. C. Molecular Design, Synthesis, and Characterization of Conjugated Polymers for Interfacing Electronic Biomedical Devices with Living Tissue. *MRS Commun* **2015**, *5* (2), 131–153. https://doi.org/10.1557/MRC.2015.17.

(9) Agache, P. G.; Monneur, C.; Leveque, J. L.; De Rigal, J. Mechanical Properties and Young's Modulus of Human Skin in Vivo. *Arch Dermatol Res* **1980**, *269* (3), 221–232. https://doi.org/10.1007/BF00406415.

(10) Koulouras, G.; Panagopoulos, A.; Rapsomaniki, M. A.; Giakoumakis, N. N.; Taraviras, S.; Lygerou, Z. EasyFRAP-web: a web-based tool for the analysis of fluorescence recovery after photobleaching data. *Nucleic Acids Res.* https://easyfrap.vmnet.upatras.gr (accessed 2024-08-07).

(11) Bali, K.; Guffick, C.; McCoy, R.; Lu, Z.; Kaminski, C. F.; Mela, I.; Owens, R. M.; van Veen, H. W. Biosensor for Multimodal Characterization of an Essential ABC Transporter for Next-Generation Antibiotic Research. *ACS Appl Mater Interfaces* **2023**, *15* (10), 12766–12776. https://doi.org/10.1021/ACSAMI.2C21556.

(12) Kang, M.; Day, C. A.; Kenworthy, A. K.; DiBenedetto, E. Simplified Equation to Extract Diffusion Coefficients from Confocal FRAP Data. *Traffic* **2012**, *13* (12), 1589–1600. https://doi.org/10.1111/TRA.12008.

(13) Chiang, P. C.; Tanady, K.; Huang, L. T.; Chao, L. Rupturing Giant Plasma Membrane Vesicles to Form Micron-Sized Supported Cell Plasma Membranes with Native Transmembrane Proteins. *Sci Reports 2017 71* **2017**, *7* (1), 1–8. https://doi.org/10.1038/s41598-017-15103-3.

(14) Richards, M. J.; Hsia, C. Y.; Singh, R. R.; Haider, H.; Kumpf, J.; Kawate, T.; Daniel, S. Membrane Protein Mobility and Orientation Preserved in Supported Bilayers Created Directly from Cell Plasma Membrane Blebs. *Langmuir* **2016**, *32* (12), 2963–2974. https://doi.org/10.1021/ACS.LANGMUIR.5B03415.

(15) Nissa, J.; Janson, P.; Berggren, M.; Simon, D. T. The Role of Relative Capacitances in Impedance Sensing with Organic Electrochemical Transistors. *Adv Electron Mater* **2021**, *7* (4), 2001173. https://doi.org/10.1002/AELM.202001173.

(16) Briand, E.; Zäch, M.; Svedhem, S.; Kasemo, B.; Petronis, S. Combined QCM-D and EIS Study of Supported Lipid Bilayer Formation and Interaction with Pore-Forming Peptides. *Analyst* **2010**, *135* (2), 343–350. https://doi.org/10.1039/B918288H.

(17) Musumeci, C.; Vagin, M.; Zeglio, E.; Ouyang, L.; Gabrielsson, R.; Inganäs, O. Organic Electrochemical Transistors from Supramolecular Complexes of Conjugated Polyelectrolyte PEDOTS. *J Mater Chem C* **2019**, *7* (10), 2987–2993. https://doi.org/10.1039/C8TC05774E.

(18) Richter, R. P.; Rodenhausen, K. B.; Eisele, N. B.; Schubert, M. Coupling Spectroscopic Ellipsometry and

Quartz Crystal Microbalance to Study Organic Films at the Solid–Liquid Interface. *Springer Ser Surf Sci* **2018**, *52*, 391–417. https://doi.org/10.1007/978-3-319-75895-4_17.

(19) Stavrinidou, E.; Gabrielsson, R.; Nilsson, K. P. R.; Singh, S. K.; Franco-Gonzalez, J. F.; Volkov, A. V.; Jonsson, M. P.; Grimoldi, A.; Elgland, M.; Zozoulenko, I. V.; Simon, D. T.; Berggren, M. In Vivo Polymerization and Manufacturing of Wires and Supercapacitors in Plants. *Proc Natl Acad Sci U S A* **2017**, *114* (11), 2807–2812. https://doi.org/10.1073/PNAS.1616456114.

(20) Dufil, G.; Parker, D.; Gerasimov, J. Y.; Nguyen, T. Q.; Berggren, M.; Stavrinidou, E. Enzyme-Assisted in Vivo Polymerisation of Conjugated Oligomer Based Conductors. *J Mater Chem B* **2020**, *8* (19), 4221–4227. https://doi.org/10.1039/D0TB00212G.

(21) Turon, X.; Rojas, O. J.; Deinhammer, R. S. Enzymatic Kinetics of Cellulose Hydrolysis: A QCM-D Study. *Langmuir* **2008**, *24* (8), 3880–3887. https://doi.org/10.1021/LA7032753.

(22) Ramanthrikkovil Variyam, A.; Stolov, M.; Feng, J.; Amdursky, N. Solid-State Molecular Protonics Devices of Solid-Supported Biological Membranes Reveal the Mechanism of Long-Range Lateral Proton Transport. *ACS Nano* **2024**, *18* (6), 5101–5112. https://doi.org/10.1021/ACSNANO.3C11990.

(23) Mantione, D.; Stavrinidou, E.; Pavlopoulou, E.; Istif, E.; Dufil, G.; Vallan, L.; Parker, D.; Brochon, C.; Cloutet, E.; Hadziioannou, G.; Berggren, M. Thiophene-Based Trimers for in Vivo Electronic Functionalization of Tissues. *ACS Appl Electron Mater* **2020**, *2* (12), 4065–4071. https://doi.org/10.1021/ACSAELM.0C00861.

(24) Volkov, A. V.; Singh, S. K.; Stavrinidou, E.; Gabrielsson, R.; Franco-Gonzalez, J. F.; Cruce, A.; Chen, W. M.; Simon, D. T.; Berggren, M.; Zozoulenko, I. V. Spectroelectrochemistry and Nature of Charge Carriers in Self-Doped Conducting Polymer. *Adv Electron Mater* **2017**, *3* (8), 1700096. https://doi.org/10.1002/AELM.201700096.
